# From College Graduate to Physician: Professional Identity Formation

**DOI:** 10.15694/mep.2020.000180.1

**Published:** 2020-08-27

**Authors:** James Ward, Virginia Randall

**Affiliations:** 1 Uniformed Services University of the Health Sciences

**Keywords:** professional identity formation, transformative experience, thematic qualitative analysis, medical students

## Abstract

This article was migrated. The article was marked as recommended.

A newly matriculated medical student must transition from college student to physician in four short years. The call has been made to help students in this transition. There is little data on how this can occur and how the system of medical education can guide students. We set out to describe and understand medical students’ process of professional identity formation (PIF) longitudinally from first-year to fourth-year students. Using a qualitative thematic analysis of 58 survey responses from the anatomy dissection laboratory (1st and 2nd year students), 78 clerkship reflective practice essays (3rd year students), and 26 survey responses to a realistic field exercise (4th year students) we developed a grounded theory: We found four developmental/transformational stages (Building, Becoming, Bridging, and Being) in PIF of a physician with the end result that the physician is confident, resilient, and embraces his/her identity. Additionally, there were three longitudinal supports identified that faculty provide: promoting self-reflection, promoting mastery of difficult tasks, and being available. Successful transition is tied to transformational experiences that with the faculty support propel the student toward PIF. These findings serve as a framework for medical educators to develop a curriculum that supports positive PIF in medical students.

## Introduction

Medical school is one of the most academically challenging educational programs one can enter. Yet through this academic rigor, a newly matriculated student must transition from undergraduate college student to physician in four short years. In recent years this process of helping students develop a professional identity of a physician has been emphasized ( Cooke et al., 2010). While most medical schools have a strong professionalism curriculum; the call was made to help students transition from simply doing the work of a physician to being a physician ( Olive and Abercrombie, 2017). There is little data on how this can occur and how the system of medical education can guide students along this journey. There are some ideas that using self-reflection and normative justification by focusing on sentinel events can help with professional identity formation ( Olive and Abercrombie, 2017). However, which sentinel events are most impactful to which student? How does one safely expose a student to these events without contributing to anxiety, burnout or compromising patient care? The literature shows that having a strong professional identity can increase resiliency that in turn reduces burn out ( Cooke et al., 2010; Cruess et al., 2014; Wald et al., 2015; McCann et al., 2013; Pendergrast and Henry, 2002).

Earlier works have cited Kegan’s 6 stage model as a starting point for developing professional identity in the health care fields ( Forsythe, 2005). The idea is that individuals progress to the point of being able to learn and think autonomously and that the medical school experience has some role in this. The interventions have not been well defined and there are differing ideas as to what interventions work best for medical students, who may vary in their progress. However, it is agreed that medical students should start the professional identity development process early in their medical school experience, and be fairly far along by graduation. The Kegan stages that are proposed to be important during medical education are stages 2-4. Stage 2 is characterized by concrete thinking and the ability to see things from other perspectives. Stage 3 individuals can develop more abstract categories and see things from multiple viewpoints. Stage 4 individuals can view things through standards and principles that are self-created ( Forsythe, 2005). There is however a lack of application of this stage model to the education system and a lack of understanding of how to encourage progression from stage to stage.

There is also the dynamic of students experiencing shame during traditional teaching, and shame is associated with students becoming withdrawn and even behavioral health issues such as substance abuse and depression ( Miles, 2019). However, is it possible to shift shame into a growth process? Is it possible to identify sentinel events that might cause shame and use those to assist in the development of professional identity? It is suggested that feeling shame shows that the student has an interest in the topic and can be used to show the student core values and opportunities for growth. The challenge becomes how does one allow shame to be experienced and intervene at the appropriate time to utilize that shame to develop the self versus allowing the shame to become destructive to the self?

Students often forgo personal needs and relationships in medical school to be successful. Indeed, often physicians sacrifice their personal wellbeing for their practice ( Pendergrast and Henry, 2002). This we would argue is poor professional identity development, the professional has eclipsed their personal. Yet this concept is only briefly addressed in medical schools and even rarely in professional practice literature.

The process of identity development is thought to happen at two levels: the individual level, dealing with one’s own psychological development; and at the collective level where an individual integrates into their new profession ( Cruess et al., 2014; Jarvis-Selinger, Pratt and Regehr, 2012).At the individual level, there have been suggestions of stages of development, however, little discussion as to how an individual progresses through those sages and how medical schools can guide students through those stages in a controlled fashion so that by graduation the student has the identity of a physician. At the social level, it is suggested that just the act of joining a new community and socializing within that community helps them move toward identifying as part of that community ( Cruess et al., 2014; Jarvis-Selinger, Pratt and Regehr, 2012; Cruess et al., 2015). Students must also learn the norms of the community and determine which community best fits them as well as which norms they will accept and reject ( Byyny, 2018). Medical school curricula allow students to “join” the community of physician through clerkships, but does simply showing up on the wards with the idea of one day being an attending physician develop a student into a physician, or is there something else that happens during this community integration process that leads to that identity development?

There has been little to show why these processes work, what the stages of the development process are and how students can be moved through these stages. We set out to describe and understand medical students’ process of professional identity formation (PIF) longitudinally from first-year to fourth-year students. Our goal was to uncover what events during medical school impact the students the most and, as they experience these events, how are they changed? Additionally, we wanted to see if there were any themes in how the faculty assisted in these events contributing to a positive PIF.

## Methods

We used qualitative thematic analysis to examine our data which consisted of 58 survey responses from the anatomy dissection laboratory (1st and 2nd year students), 78 clerkship reflective practice essays (3rd year students), and 26 survey responses to an intense and realistic battlefield exercise (4th year students). We adhered to careful line-by-line coding so as not to lose the intent of the student’s voice and frequent re-reading with constant comparison to previous codes and quotes. We discussed each line and code until we reached agreement. We used NVivo 11© to record 115 codes.

We then independently arranged our codes into themes and discussed our arrangement until we reached agreement. As we examined our themes, we found the emergence of a theory, grounded in these themes.

The co-investigators represent different professional backgrounds (social work vs chemistry major in college, different points in a medical career, and represent different generations of physicians’ assessments of professionalism.) We believe these differences in perspective leant depth to our analysis.

## Results/Analysis

**Figure 1.**
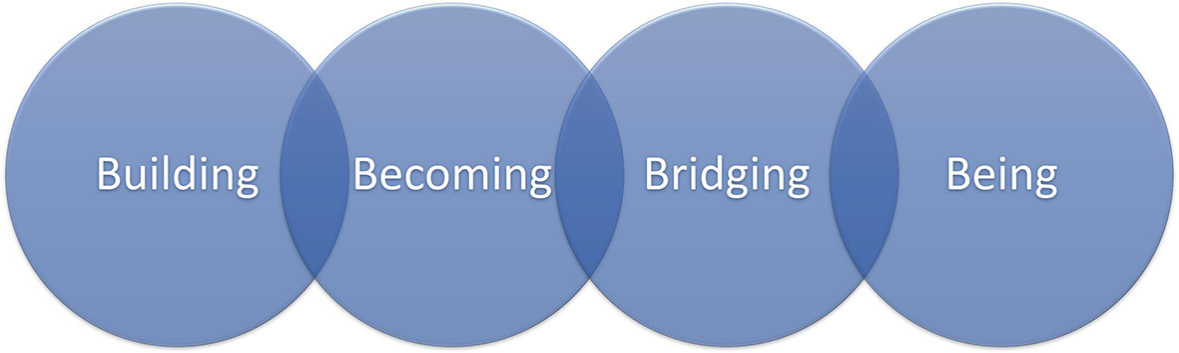
Stages in Professional Development of a Physician

Emergent theory: There are four developmental stages in Professional Identity Formation (PIF) of a physician with the end result that the physician is confident and embraces his/her identity. ( Figure 1)

**Building stage:** These are events, realizations, and experiences that occur early in the process of PIF that help set the stage for students to practice becoming a physician.

**Becoming stage:** in this stage, students have experiences and realizations that demonstrate that they are starting to think and act like physicians even though they have not fully integrated being a physician into their identity.

**Bridging stage:** these are experiences that help shift the student into the stage where they begin to feel like a physician and start to incorporate physician into their identity.

**Being stage:** this is the stage where students make the ontological shift to identifying as a physician.

With the successful progression through the stages, we see individuals who find joy in being a physician. They are more resilient and resistant to burnout. Each stage builds on previous stages and transformative events allow progression through the stages. The transformative events were seen as occurring in the bridging stage.

We found three longitudinal factors that supported students as they traversed the role of college graduate to physician. These factors require the active engagement of faculty who are aware of and sympathize with the struggles of students.

## Discussion

**Figure 2.**
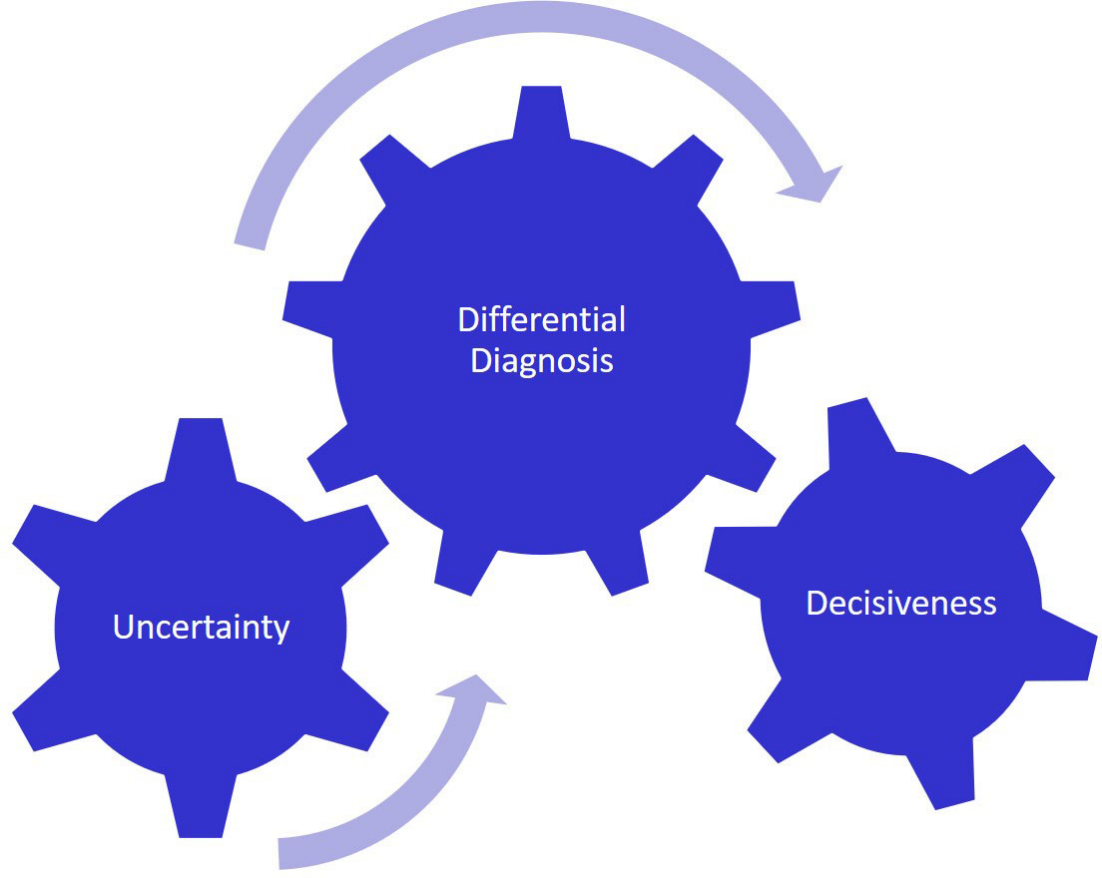
Differential Diagnosis

The transformative event appears to bridge concepts seen in the building or becoming stage with concepts seen in the being stage of PIF. The four transformative events were identified as: differential diagnosis, experiencing being a member of a team, knowledge itself isn’t enough, and dealing with difficult situations. ( Figure 2)

In addition to linking decisiveness with uncertainty, differential diagnosis was seen as linking preclinical knowledge with the reality of patient treatment.

“ *Taken another way, uncertainty is perhaps the most important concept that I have learned with respect to thinking like a physician. I see this first and foremost in the context of the differential diagnosis. When this concept was first fully introduced in Pathology Small Groups, I thought of the differential as an exercise where you ‘won’ if the correct diagnosis was at the top of your list. While I still like it when I am able to nail a diagnosis, I have come to have a greater appreciation for the differential as a tool for when things are less certain.”*

**Figure 3.**
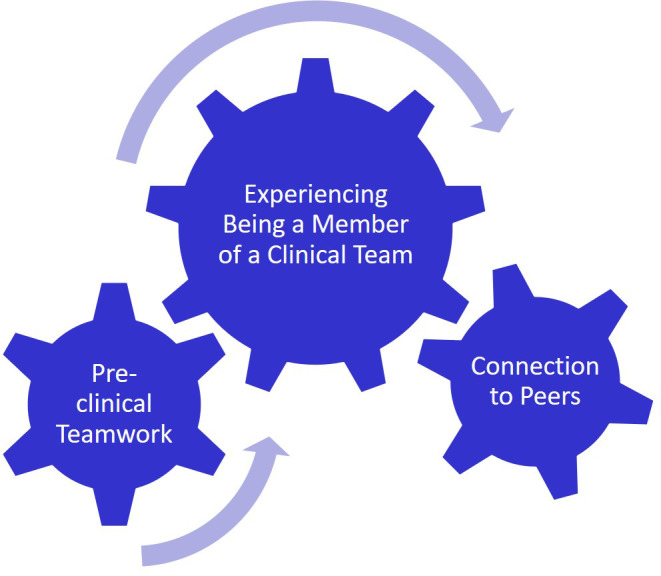
Experiencing being a Member of a Clinical Team

While working on a team is not a new concept to USU students, being part of a *clinical* team is new to most and the team operates differently than other teams they may have been on. ( Figure 3) This experience connects the communication skills, leadership and followership skills learned in the pre-clinical setting to teams of peers on a professional level in the patient care environment. This also served to educate students on the roles of the different team members as well as show the reality of healthcare being delivered in a team-driven environment.

“ *After all, medical doctors are the ones who must endure extensive hours of studying and learning to be able to provide the most up-to-date care for the patients. However, I realized that quality care is not possible without involvement of the entire care team including nurses, therapists and technicians. Without their meticulous care and passion for the patients, the prescriptions and treatment plans from medical doctors often remain inadequate.”*

**Figure 4.**
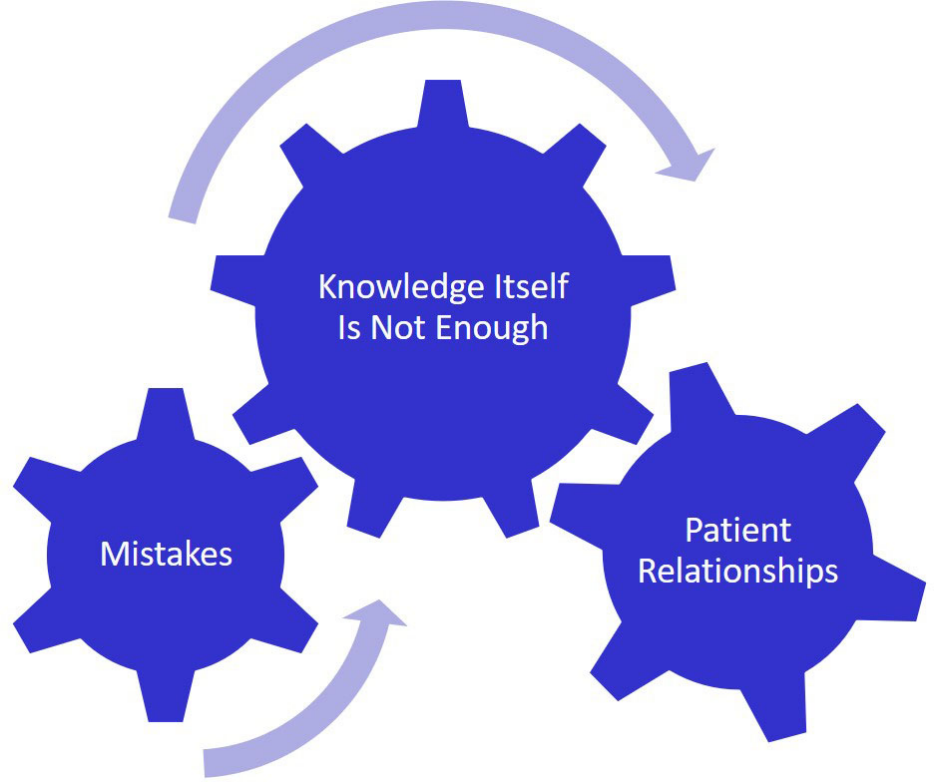
Knowledge Itself is Not Enough

The realization that Knowledge ( Figure 4) itself isn’t enough to provide high-quality patient care is something that students experience during clerkships. They realize that other factors play into patient outcomes; things such as doctor-patient relationships, patient buy-in and communication skills. This initial idea of knowledge being all that is needed appears to come from a pre-med culture where testing and academic success are rewarded.

“In pre-clerkship, I was always self-centered, caring most importantly on whether I could memorize concepts and understand enough in order to pass each shelf exam. When I started the clerkship year, at first my highest priority was to study each and every day for the shelf at the end of the block. However, as I began to have more and more patient encounters, it was very apparent to me that the medical knowledge that I was being tested on has certain and important applications. I was no longer studying for the test, I was studying in order to better take care of real human beings who had the same sorrows, joys, and anxieties that I have.”

**Figure 5.**
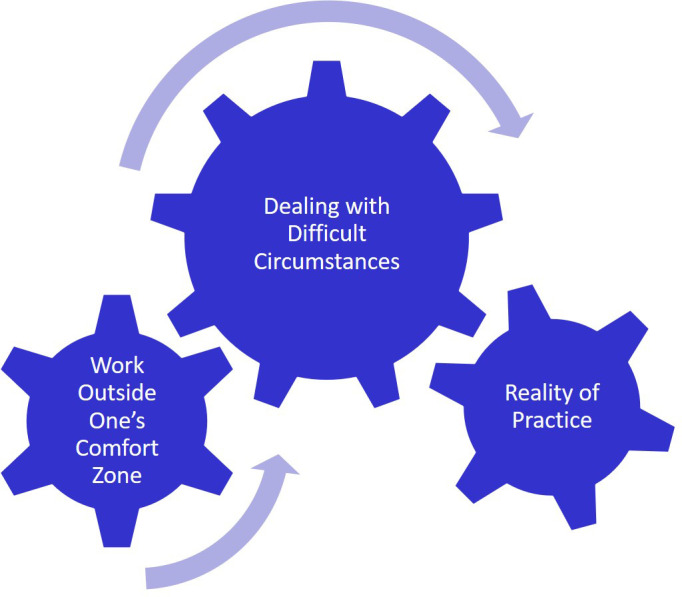
Dealing with Difficult Situations

Learning to be comfortable and confident in difficult situations ( Figure 5) starts in the anatomy lab. As students enter the clinical environment they realize that they will continually face these challenges and must continue to embrace them. The challenges vary in magnitude and in degree for students.

“While sadness has definitely permeated my thoughts, the death of these children has also motivated me to work harder.”

“One day I may be the only person to see a patient, and it will be my responsibility to make sure that I do not miss any potentially devastating diseases.”

**Figure 6.**
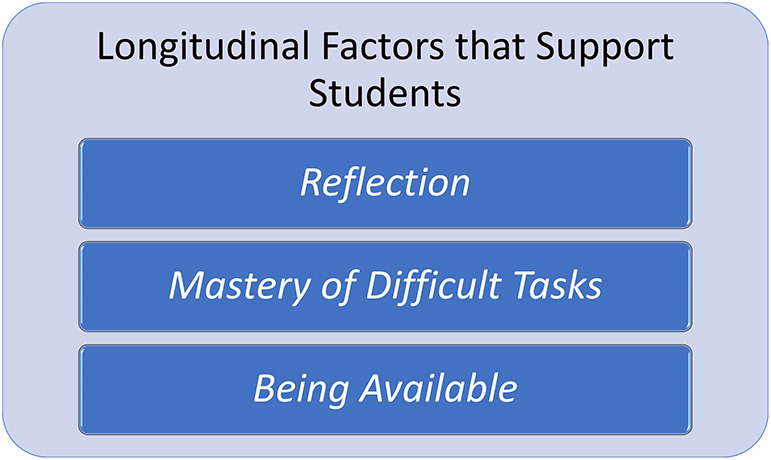
Major Longitudinal Supports Provided by Faculty

Major supports provided by faculty were also identified: promotion of self-reflection, scaffolding for mastery of difficult tasks and being available. These supports were seen as imperative in helping students successfully transition through the process of professional identity development. ( Figure 6).

### Reflection

Self-reflection that leads to increased self-awareness allows the student to more comfortably move into professional identity formation. Self-reflection has been shown in multiple studies to be a method of addressing professionalism as the student learns to evaluate his own behaviors, attitudes, and biases in light of standards in the practice of medicine. Requiring written and/or oral self-reflection, with prompt questions appropriate to the students’ setting, can set up the mechanism and habit of reflection. For instance, having anatomy students write about their first reaction to seeing the cadaver feels pertinent to the student, and allows them to explore the range of emotions typically encountered in the anatomy laboratory. Having an opportunity to discuss the topic with faculty helps the student understand their own reactions. Further, it is useful for students to share their reflections, as often the student is feeling alone and isolated, whereas many other students are sharing those same feelings.

### Mastery of Difficult Tasks

New and frequently difficult tasks for students include cognitive and psychomotor skills. Cognitive tasks, such as patient interviewing, creating a differential diagnosis, researching medical literature for evidence, presenting a patient on ward rounds or morning report - these are all cognitive tasks that the student must learn, but not all at once. The elements of the tasks can be scaffolded by the preceptor so that the student achieves mastery at an early step and feels confident to move to the next step. For instance, presenting a patient in morning report can be daunting. However, starting with having the student write out the presentation, practice in private with the preceptor, engage a few other students to ask questions about the presentation, present in a small forum such as student lunch conference, moving to presenting on rounds, then finally presenting in morning report makes this doable. It is crucial that the preceptor engage the student with questions and comments from the audience that might be anticipated, that the preceptor gives precise constructive feedback to improve the presentation and guide the student to relevant medical literature.

A difficult psychomotor skill might be starting an IV. This begins with having the student watch a seasoned health care provider (nurse, physician, resident) start an IV while talking about the process with the student. The student should observe consent discussions with the patient. The student should be instructed on how the equipment is selected and used, how the vein is found, puncturing the skin and getting the needle into the vein. In some instances, there will be a blood draw through the needle prior to starting the IV. After a few demonstrations, the student can begin by talking with the patient including informing the patient that he is a medical student under the direct supervision of a faculty member; selecting the equipment; finding the vein; etc. If at any point the student seems to hesitate, the preceptor can encourage and further instruct the student; such as, anchoring a rolling vein, when the needle tip is against a valve, etc. Again, precise constructive and laudatory feedback is important to encourage the student into a feeling of mastery.

A feeling of mastery is vital to the student achieving increased confidence. This mastery and confidence can frame elements of the professional identity (such as, I am good at starting IVs). While not dramatic statements, these are important building blocks of PIF without which the student may struggle with self-doubt and cynicism.

### Being Available

Through all 4 years of medical school, the availability of suitable faculty to provide support to the student is vital. Discussion individually or in groups that encourage students through difficult circumstances, such as 4 high stakes exams in one week, can offer students encouragement. Individual discussions with students can find out about their family circumstances, prior employment, health issues, that may impact how the student perceives the role of physician and how they will approach work-life balance issues.

Being available also includes being aware of the strong influence of role models on students’ PIF. Faculty consciously or unconsciously model behaviors from the negative hidden curriculum (being rude to subordinate staff) and from appropriate professionalism in the hidden curriculum (promptly returning patient phone calls) Being aware of one’s role modeling, especially in patient relationships, also triggers reflection on the part of faculty and can improve their attention to their own behavior.

**Limitations and Strengths:** This study is limited in its generalizability by being conducted at only one medical school, a military medical school. It is possible that military traditions have influenced our results. Its strength lies in the large amount of data we were able to access.

## Conclusion

During the process of medical education, in addition to the academic learning that must take place, there is a need to develop a professional identity as a physician. Our research has identified four phases in professional identity development: building, becoming, bridging, and being. While progressing through the stages, students rely on the support of the faculty to help them frame experiences as opportunities to grow. The three most important faculty supports were identified as reflection, mastery of difficult tasks, and being available. During the Building stage, students have transformative experiences that tie the prior stages into the identity of a physician. These experiences were identified as creating a differential diagnosis, experiencing being a member of a team, the realization that knowledge itself is not enough, and dealing with difficult situations.

## Take Home Messages

• Professional identity formation is a significant goal of medical school.
• Professional identity formation involves several stages from novice to professional. These stages include transformative experiences, in which the student comes to experience a new self, that of a physician.
• Faculty support identity formation by providing for self-reflection, scaffolding difficult tasks, and being available for the student.

## Notes On Contributors

Lieutenant James Ward, Medical Doctor, United States Navy, is newly graduated from Uniformed Services University of the Health Sciences (class 2020) and was deployed to Fort Meade, Maryland, at the beginning of the Covid-19 pandemic. He completed an Masters of Social Work prior to coming to medical school and will begin a Family Medicine Residency at Camp Pendelton, California. in July 2020. Lieutenant Ward completed the research, manuscript preparation, and a poster as his senior year research rotation.

Virginia Randall, Medical Doctor, Master of Public Health, was an active duty pediatrician in the United States Army for 30 years prior to coming to Uniformed Services University of the Health Sciences, where she has been teaching and doing research with medical students for the past 15 years. Doctor Randall served as mentor for Lieutenant Ward, including co-developing coding for the qualitative analysis and editing the manuscript. ORCID: https://orcid.org/0000-0003-2944-9015

## Declarations

The author has declared that there are no conflicts of interest.

## Ethics Statement

Uniformed Services University (USU) Human Research Protections Program (HRPP) Amendment 12 Approval for Exempt Protocol PED-86-4343 eIRB ref. #913361. The study is educational research and is no more than minimal risk.

## External Funding

This article has not had any External Funding
