# Supplementary material for: From College Graduate to Physician: Professional Identity Formation
Source: MedEdPublish (2016). 2020 Aug 27;9:180. [Version 1] doi: 10.15694/mep.2020.000180.1 (PMC10702648; doi:10.15694/mep.2020.000180.1)
Supplement: Peer review history

# Reviewer report: Reviewer response for version 1

**DOI:** 10.21956/mep.20063.r30803

**Published:** 2020-09-10

**Authors:** P Ravi Shankar

**Affiliations:** 1 American International Medical University

This review has been migrated. The reviewer awarded 4 stars out of 5 I enjoyed reading this article on professional identity formation among medical students. The process is well-described and documented. There may be differences in the process of professional identity formation (PIF) between the US where students complete a four-year MD program after graduation as opposed to countries like India where they enter medical school earlier (after twelve years of schooling) and complete a five and half year undergraduate medical program. As the authors mentions there may be differences in PIF between students at a military medical school and those in other institutions. In a military medical school, students may already have an identity as a member of the armed forces on to which that of a physician is incorporated. This is a topic worthy of further detailed investigation.

Reviewer Expertise:

NA

No decision status is available

# Reviewer report: Reviewer response for version 1

**DOI:** 10.21956/mep.20063.r30800

**Published:** 2020-09-06

**Authors:** Shamaila ijaz

**Affiliations:** 1 Lisca

This review has been migrated. The reviewer awarded 4 stars out of 5 The paper has explained in detail the transition from a student to physician. The abstract is well defined. Introduction has latest literature and theories. The results are accurately defined though number of sample could be expanded. The discussion shows that it should be inculcated as curriculum in medical education so that students should have a smooth transition rather than finding difficulty or passing through trail and error stages.

Reviewer Expertise:

NA

No decision status is available

# Reviewer report: Reviewer response for version 1

**DOI:** 10.21956/mep.20063.r30801

**Published:** 2020-09-03

**Authors:** Wai Ching Lee

**Affiliations:** 1 Sengkang General Hospital

This review has been migrated. The reviewer awarded 4 stars out of 5 Very enlightening paper and very well explained and done in the discussion part. Agree with the 1st comment to make the methodology and perhaps the list of interview questions available as well to build the transferability of this study

Reviewer Expertise:

NA

No decision status is available

# Reviewer report: Reviewer response for version 1

**DOI:** 10.21956/mep.20063.r30802

**Published:** 2020-08-27

**Authors:** Trevor Gibbs

**Affiliations:** 1 AMEE

This review has been migrated. The reviewer awarded 4 stars out of 5 I very much enjoyed reading this paper, and learned much from my reading. I felt that it was well written, well explained and covers a very important area in the life of an health professional. I did want to ask questions about further explaining the methodologies used, the intricacies of each and the difference with each group of students, but wanting to inquire further IU take to be a good sign in this paper. Although it does appear to be US-centric including the references, I think that it does have transferability qualities.I would recommend it to all involved in curriculum development and professional identity formation

Reviewer Expertise:

NA

No decision status is available
